# Ready and waiting to go

**DOI:** 10.7554/eLife.85080

**Published:** 2023-01-06

**Authors:** Laura Rivino, Linda Wooldridge

**Affiliations:** 1 School of Cellular and Molecular Medicine, Faculty of Life Sciences, University of Bristol Bristol United Kingdom; 2 Bristol Veterinary School, Faculty of Health Sciences, University of Bristol Bristol United Kingdom

**Keywords:** heterologous immunity, cross-reactivity, T cells, HCMV, SARS-CoV-2, Human

## Abstract

Some T cells that have been activated by a herpesvirus can also respond to SARS-CoV-2, even if the original herpesvirus infection happened before the COVID-19 pandemic.

**Related research article** Pothast CR, Dijkland RC, Thaler M, Hagedoorn RS, Kester MGD, Wouters AK, Hiemstra PS, van Hemert MJ, Gras S, Frederik Falkenburg JH, Heemskerk MHM. 2022. SARS-CoV-2-specific CD4+ and CD8+ T cell responses can originate from cross-reactive CMV-specific T cells. *eLife* **11**:e82050. doi: 10.7554/eLife.82050.

Cells called T cells play an important role in protecting the body against infection by removing pathogens that may cause harm. Two major types of T cell are involved in the response to a viral infection. Both become activated when their receptors recognize short peptides from viral proteins called ‘epitopes’: CD8 T cells directly attack infected cells, whereas CD4 T cells help other immune cells (called B cells) to produce antibodies. Once the infection has been eliminated, some of these CD8 and CD4 T cells survive in the body as long-lived memory T cells which can immediately respond if the virus invades again.

Previous studies found that some blood samples taken before the COVID-19 pandemic already contained T cells that could recognize the SARS-CoV-2 virus (Grifoni et al., 2020; Le Bert et al., 2020). However, researchers still do not fully understand how these T cells arose, or how they impact immunity and disease outcomes for COVID-19 patients.

One possibility is that these pre-existing T cells arose due to a phenomenon called heterologous immunity (Welsh et al., 2010). This is when CD4 and CD8 T cells activated by a specific pathogen ‘cross-react’ and respond to epitopes from a different virus (Mason, 1998). It was previously thought that coronaviruses already circulating in the population before the pandemic were responsible for the existence of some T cells that could recognise SARS-CoV-2 (Grifoni et al., 2020; Swadling et al., 2022). Now, in eLife, Cilia Pothast (Leiden University Medical Center), Mirjam Heemskerk (also at Leiden) and colleagues report that another group of viruses may have also been involved (Pothast et al., 2022).

The team hypothesised that some of the T cells specific to SARS-CoV-2 had been activated by a herpesvirus called human cytomegalovirus (HCMV). This pathogen is highly prevalent in the population and has also been linked to changes in the severity of COVID-19 symptoms (Alanio et al., 2022). To investigate, they stimulated pre-pandemic blood samples with different segments of SARS-CoV-2 proteins. This led them to discover a population of ‘cross-reactive’ CD4 and CD8 T cells that can recognize epitopes from both SARS-CoV-2 and HCMV (Figure 1).

**Figure 1.**
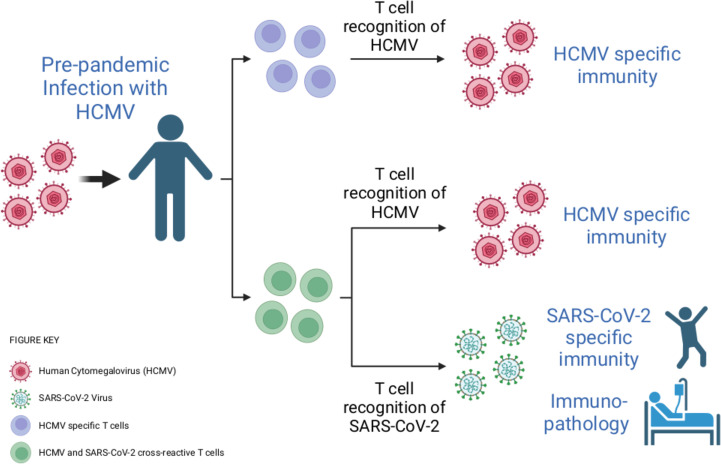
Infection with human cytomegalovirus (HCMV) can stimulate T cells that can recognise SARS-CoV-2. When individuals are infected with HCMV (virus shown in pink), the population of T cells that can detect this virus expands (T cells shown here in purple). Cross-reactivity is a well-known feature of the immune response. Through this process, HCMV infection can activate T cells (shown here in green) that can recognise both HCMV and another pathogen – including the SARS-CoV-2 virus, even if the HCMV infection happened before the COVID-19 pandemic. These cross-reactive T cells may be able to contribute to the immunity of an individual to SARS-CoV-2, as well as to how COVID-19 affects their body.

Image credit: Created with BioRender.com.

Pothast et al. found that this cross-reactivity was due to a T cell receptor that is expressed in multiple individuals. However, there are very few similarities between the amino acid sequences of the SARS-CoV-2 and the HCMV epitopes, bringing into question how this T cell receptor can detect both viruses. It may be possible to explain the molecular basis for this observation by solving crystal structures of this T cell receptor in complex with either the presented HCMV or SARS-CoV-2 epitopes.

Further experiments then revealed that the cross-reactive T cells limit the replication of SARS-CoV-2 in vitro when the virus is present at low levels. However, the cross-reactive T cells did not appear to have an activated phenotype in patients with severe COVID-19. This might be because individuals included in this study were over 60 years of age, and HCMV-specific T cells do not work as well as people get older (Ouyang et al., 2004).

It has been suggested that heterologous immunity may play a beneficial role in protective immunity (Welsh et al., 2010). This is consistent with a recent study showing that T cells which cross-react with SARS-CoV-2 are associated with abortive infections (when the virus fails to spread to other cells) and asymptomatic cases of COVID-19 (Swadling et al., 2022). These pre-existing T cells may also enhance a person’s response to vaccines (Loyal et al., 2021). However, heterologous immunity is a double-edged sword, as it can also increase the severity of some viral infections. For example, in dengue infections, cross-reactive antibodies and T cells can result in an immune response that is harmful to the body (Welsh et al., 2010; Screaton et al., 2015).

Further studies are needed to establish whether other pathogens (including bacteria) can stimulate T cells capable of recognising epitopes from SARS-CoV-2. In addition, studies with larger cohorts of vaccinated individuals and patients with mild or severe COVID-19 are required to define the role that these cross-reactive T cells play in protective immunity, in response to vaccination, and in disease pathology.
